# On the optimal control of coronavirus (2019-nCov) mathematical model; a numerical approach

**DOI:** 10.1186/s13662-020-02982-6

**Published:** 2020-09-25

**Authors:** N. H. Sweilam, S. M. Al-Mekhlafi, A. O. Albalawi, D. Baleanu

**Affiliations:** 1 Faculty of Science, Department of Mathematics, Cairo University, Giza, Egypt; 2 Faculty of Education, Department of Mathematics, Sana’a University, Sana’a, Yemen; 3 Department of Mathematics, Faculty of Science, Shaqra University, Riyadh, Kingdom of Saudi Arabia; 4 Department of Mathematics, Cankaya University, Ankara, Turkey; 5 Institute of Space Sciences, Magurele-Bucharest, Romania

**Keywords:** Coronavirus diseases, Fractional order optimal control problems, Grünwald–Letnikov nonstandard weighted average finite difference method

## Abstract

In this paper, a novel coronavirus (2019-nCov) mathematical model with modified parameters is presented. This model consists of six nonlinear fractional order differential equations. Optimal control of the suggested model is the main objective of this work. Two control variables are presented in this model to minimize the population number of infected and asymptotically infected people. Necessary optimality conditions are derived. The Grünwald–Letnikov nonstandard weighted average finite difference method is constructed for simulating the proposed optimal control system. The stability of the proposed method is proved. In order to validate the theoretical results, numerical simulations and comparative studies are given.

## Introduction

The well-known coronavirus disease (COVID-19) pandemic can be consider as one of the serious pandemic diseases all over the world, for more details, see [1, 2]. The spread of this disease has serious impact on human society and health. The modeling study of infectious diseases is very useful in making strategies to control this disease. Recently, many interesting papers on modeling the coronavirus have appeared, see for example [3–7].

In general, mathematical models involving the known ordinary differentiation could be used to capture dynamical systems of infectious disease, when only initial conditions are used to predict future behaviors of the spread. However, when the situation is unpredictable, which is the case of COVID-19, due to uncertainties associated with the pandemic, ordinal derivatives and their associated integral operators show deficiency. The fractional order differential equation (FODE) models seem more consistent with the real phenomena than the integer order models [8–13].

Moreover, one of the new topics in mathematics is the fractional optimal control (FOC). FOC can be defined using a variety of fractional derivative definitions. Riemann–Liouville and Caputo fractional derivatives [14–18] can be considered the most important fractional derivative definitions. Sweilam and Al-Mekhlafi introduced some numerical studies for FOC, for more details, see [19–21].

The main contribution of this work is to develop a numerical scheme to provide approximate solutions for the fractional optimal control problems (FOCPs). We consider the mathematical model in [4] with modified parameters. The fractional order derivatives are defined here in the Caputo sense. Moreover, we introduce two control variables, $u_{p} ( t )$ and $u_{ap} ( t )$, in order to minimize the number of the infected and the asymptotically infected. The Grünwald–Letnikov nonstandard weighted average finite difference method (GL-NWAFDM) is established to simulate the obtained fractional order system.

To the best of our knowledge, the fractional optimal control for coronavirus (2019-nCoV) mathematical model with GL-NWAFDM has never been explored.

This paper is organized as follows: The basic mathematical formulas are introduced in Sect. 2. The proposed model with FO and two controls is presented in Sect. 3. In Sect. 4, the formulation of the optimal control problem and the necessary optimality conditions are derived. In Sect. 5, the new numerical method GL-NWAFDM and the stability analysis are introduced. Numerical simulations are discussed in Sect. 6. Finally, the conclusions are presented in Sect. 7.

## Preliminaries and notations

In this section, we recall some important definitions of fractional calculus used throughout the remaining sections of this paper. The fractional order derivative in the Caputo sense can be defined as follows [22]:

$$ {}_{a}^{c} D_{t}^{\alpha } f ( t )= \frac{1}{\Gamma ( n-\alpha )} \int _{a}^{t} ( t-\tau )^{n-\alpha - 1} f^{( n )}\,d\tau , $$

where Γ is the Euler gamma function and $0< \alpha <1$.

The discretization fractional derivative is given by the Grünwald–Letnikov approach [23]:

1 $$ {}_{a}^{C} D_{t}^{\alpha } y ( t ) \mid _{t = t^{n}} = \frac{1}{\triangle t^{\alpha }} \Biggl( y_{n+ 1} - \sum _{i =1}^{n+ 1} \mu _{i} y_{n+ 1 -i} - q_{n+ 1} y_{0} \Biggr), $$

where $N_{n}$ is a natural number and the coordinate of each mesh point is $t^{n} = n \triangle t $, $n =1,2,\ldots, N_{n}$, $\triangle t = \frac{T_{f}}{N_{n}}$, $\mu_{i}={\left(-1\right)}^{i-1}\left(\begin{array}{c} \alpha \\ i \end{array}\right)$, $\mu _{1} = \alpha $, $q_{i} = \frac{i^{\alpha }}{\Gamma (1 -\alpha )}$, and $i =1,2,\ldots, n+ 1$. Additionally, let us assume that [24]

$$\begin{aligned}& 0< \mu _{i+ 1} < \mu _{i} < \cdots< \mu _{1} = \alpha < 1, \\& 0< q_{i+ 1} < q_{i} < \cdots< q_{1} = \frac{1}{\Gamma (1 -\alpha )}. \end{aligned}$$

## Fractional order model of coronavirus with control

Herein, we consider the new mathematical model of coronavirus given in [4] with modified parameters. Two controls, $u_{p}$, $u_{ap}$, are added to health care such as isolating patients in private health rooms and providing respirators and giving them treatments soothing regularly. This model consists of six nonlinear ordinary differential equations. Moreover, Table 1 describes the state variables and Table 2 describes the parameters. It is important to notice that the parameters depend on the fractional order *α*. To make the system consistent in the physical sense and more consistent with the reality, we must make sure that the right-hand sides of these equations have the same dimensions, for more details, see [15]. Let us assume that $N_{p}$ is a constant. The modified model is then represented by a system of fractional order differential equations:

2 $$\begin{aligned}& {}_{a}^{C} D_{t}^{\alpha } S_{p} = \pi _{p}^{\alpha } - \mu _{p}^{\alpha } S_{p} - \frac{\eta _{p}^{\alpha } ( I_{p} + \psi ^{\alpha } A_{p} )}{N_{p}} - \eta _{w}^{\alpha } S_{p} M, \\& {}_{a}^{C} D_{t}^{\alpha } E_{p} = \frac{\eta _{p}^{\alpha } ( I_{p} + \psi ^{\alpha } A_{p} )}{N_{p}} + \eta _{w}^{\alpha } S_{p} M- (1 - \theta _{p} ) w_{p}^{\alpha } E_{p} - \theta _{p}^{\alpha } \rho _{p}^{\alpha } E_{p}, \\& {}_{a}^{C} D_{t}^{\alpha } I_{p} =\bigl(1 - \theta _{p}^{\alpha } \bigr) w_{p}^{\alpha } E_{p} - \bigl( \tau _{p}^{\alpha } + \epsilon _{1} u_{p} + \mu _{p}^{\alpha } \bigr) I_{p}, \\& {}_{a}^{C} D_{t}^{\alpha } A_{p} = \theta _{p}^{\alpha } \rho _{p}^{\alpha } E_{p} - \bigl( \tau _{ap}^{\alpha } + \epsilon _{2} u_{ap} + \mu _{p}^{\alpha } \bigr) A_{p}, \\& {}_{a}^{C} D_{t}^{\alpha } R_{p} = \tau _{p}^{\alpha } I_{p} + \epsilon _{1} u_{p} I_{p} + \tau _{ap}^{\alpha } A_{p} + \epsilon _{2} u_{ap} A_{p} - \mu _{p}^{\alpha } R_{p}, \\& {}_{a}^{C} D_{t}^{\alpha } M = \varrho _{p}^{\alpha } I_{p} + \varpi _{p}^{\alpha } A_{p} - \pi ^{\alpha } M. \end{aligned}$$

**Table 1:** The variables of system (2) [4]

Variable	Description
$S_{p}$	Susceptible humans
$E_{p}$	Exposed humans
$I_{p}$	Infectious humans
$A_{p}$	Asymptotically infected
$R_{p}$	Recovered humans
*M*	The reservoir or the seafood place or market
$N_{p}$	The total population
	$N_{p} = S_{p} + E_{p} + I_{p} + R_{p}$

**Table 2:** The parameter values for COVID model [4]

Parameter	Description	Value (per $da y^{\alpha } $)
$\pi _{p}^{\alpha } $	Birth rate	$( \mu _{p} \times N_{p} (0) )^{\alpha } $
$\mu _{p}^{\alpha } $	Natural mortality rate	$( \frac{1}{76.79 \times 365} )^{\alpha } $
$\eta _{p}^{\alpha } $	Contact rate	$(0.05 )^{\alpha } $
$\psi ^{\alpha } $	Transmissibility multiple	$(0.02 )^{\alpha } $
$\eta _{w}^{\alpha } $	Disease transmission coefficient	$(0.000001231 )^{\alpha } $
$\theta _{p}^{\alpha } $	The proportion of asymptomatic infection	$(0.1243 )^{\alpha } $
$w_{p}^{\alpha } $	Incubation period	$(0.00047876 )^{\alpha } $
$\rho _{p}^{\alpha } $	Incubation period	$(0.005 )^{\alpha } $
$\tau _{p}^{\alpha } $	Recovery rate of $I_{p}$	$(0.09871 )^{\alpha } $
$\tau _{ap}^{\alpha } $	Recovery rate of $A_{p}$	$(0.854302 )^{\alpha } $
$\varrho _{\alpha } $	M-virus contribution by $I_{p}$	$(0.000398 )^{\alpha } $
$\varpi _{p}^{\alpha } $	M-virus contribution by $A_{p}$	$(0.001 )^{\alpha } $
$\pi ^{\alpha } $	Virus removing rate from *M*	$(0.01 )^{\alpha } $

The existence and uniqueness of the solutions of (2) follow from the results given in [25]. The feasible region for model (2) is given by

$$ \Omega = \biggl\{ S_{p}, I_{p}, E_{p}, R_{p}, A_{p} \in \mathbb{R}^{5}: N_{p} ( t ) \leq \frac{\pi _{p}^{\alpha }}{\mu _{p}^{\alpha }}, M\in \mathbb{R}^{+} \biggr\} . $$

The basic reproduction number of the proposed model (2) is given as follows [4]:

3 $$ R_{0} = R_{1} + R_{2}, $$

where

$$\begin{aligned}& R_{1} = \frac{\theta _{p}^{\alpha } \rho _{p}^{\alpha } ( \pi ^{\alpha } \varpi _{p}^{\alpha } \eta _{p}^{\alpha } \mu _{p}^{\alpha } + \pi _{p}^{\alpha } \psi _{p}^{\alpha } \eta _{w}^{\alpha } )}{\pi ^{\alpha } \mu _{p}^{\alpha } ( \tau _{ap}^{\alpha } + \mu _{p}^{\alpha } )( \theta _{p}^{\alpha } \rho _{p}^{\alpha } + (1 - \theta _{p}^{\alpha } ) w_{p}^{\alpha } + \mu _{p}^{\alpha } )}, \\& R_{2} = \frac{(1 - \theta _{p}^{\alpha } ) w_{p}^{\alpha } ( \pi ^{\alpha } \eta _{p}^{\alpha } \mu _{p}^{\alpha } + \pi _{p}^{\alpha } \varrho _{p}^{\alpha } \eta _{w}^{\alpha } )}{\pi ^{\alpha } \mu _{p} ( \tau _{ap}^{\alpha } + \mu _{p}^{\alpha } )( \theta _{p}^{\alpha } \rho _{p}^{\alpha } + (1 - \theta _{p} ) w_{p}^{\alpha } + \mu _{p}^{\alpha } )}. \end{aligned}$$

The endemic threshold is given at $R_{0} =1$, the disease will die out when $R_{0} <1$, and the endemic case when $R_{0} <1$, for more details, see [26]. In this work we consider $R_{0} >1$.

## The FOCPs

Consider system (2) in $\mathbb{R}^{6}$, let

$$\begin{aligned} U =&\bigl\{ \bigl( u_{p} (\cdot), u_{ap} (\cdot)\bigr) \mid u_{p}, u_{ap}\mbox{ are Lebsegue measurable on } [0,1], \\ &{}0 \leq u_{p} (\cdot), u_{ap} (\cdot) \leq 1, \forall t\in [0, T_{f} ]\bigr\} \end{aligned}$$

be the admissible control set. We define the objective functional as follows:

4 $$ J ( u_{p}, u_{ap} )= \int _{0}^{T_{f}} \biggl( I_{p} ( t ) + A_{p} ( t ) + \frac{B_{1}}{2} u_{p} ( t ) + \frac{B_{2}}{2} u_{ap} ( t )\biggr)\,dt. $$

Now, the goal is to evaluate $u_{p}$, $u_{ap}$ such that the following functional

5 $$ J ( u_{p}, u_{ap} )= \int _{0}^{T_{f}} \eta ( S_{p}, E_{p}, I_{p}, A_{p}, R_{p}, M, u_{p}, u_{ap}, t )\,dt $$

is minimum, subject to the constraints

6 $$ {}_{a}^{C} D_{t}^{\alpha } \Psi _{j} = \xi _{i}, $$

where

$$\begin{aligned}& \xi _{i} = \xi _{i} ( S_{p}, E_{p}, I_{p}, A_{p}, R_{p}, M, u_{p}, u_{ap}, t ),\quad i =1,\ldots,6, \\& \Psi _{j} =\{ S_{p}, E_{p}, I_{p}, A_{p}, R_{p}, M \ j =1,\ldots,6\}, \end{aligned}$$

and satisfying the initial conditions

$$\begin{aligned}& \Psi _{1} (0)= S_{p 0},\qquad \Psi _{2} (0)= E_{p 0},\qquad \Psi _{3} (0)= I_{p 0}, \\& \Psi _{4} (0)= A_{p 0},\qquad \Psi _{5} (0)= R_{p 0},\qquad \Psi _{6} (0)= M_{0}. \end{aligned}$$

We use a kind of Pontryagin maximum principle in the fractional order case, this idea in fraction is given by Agrawal in [18].

Consider a modified cost functional as follows [19]:

7 $$\begin{aligned} \tilde{J} =& \int _{0}^{T_{f}} \Biggl[ H ( S_{p}, E_{p}, I_{p}, A_{p}, R_{p}, M, u_{p}, u_{ap}, t ) \\ &{}- \sum_{i =1}^{6} \lambda _{i} \xi _{i} ( S_{p}, E_{p}, I_{p}, A_{p}, R_{p}, M, u_{p}, u_{ap}, t )\Biggr]\,dt. \end{aligned}$$

The Hamiltonian is defined as follows:

8 $$\begin{aligned}& H ( S_{p}, E_{p}, I_{p}, A_{p}, R_{p}, M, u_{p}, u_{ap}, t ) \\& \quad = \eta ( S_{p}, E_{p}, I_{p}, A_{p}, R_{p}, M, u_{p}, u_{ap}, \lambda _{i}, t ) \\& \qquad {}+ \sum_{i =1}^{6} \lambda _{i} \xi _{i} ( S_{p}, E_{p}, I_{p}, A_{p}, R_{p}, M, u_{p}, u_{ap}, t ). \end{aligned}$$

From (7) and (8), we have the FOPC necessary conditions:

9 $$ {}_{t}^{C} D_{t_{f}}^{\alpha } \lambda _{\iota } = \frac{\partial H}{\partial \vartheta _{\iota }},\quad \iota =1,\ldots,6, $$

where

10 $$\begin{aligned}& \vartheta _{\iota } =\{ S_{p}, E_{p}, I_{p}, A_{p}, R_{p}, M, u_{p}, u_{ap}, t, \iota =1,\ldots,6\}, \\& 0= \frac{\partial H}{\partial u_{k}},\quad k = p, ap, \end{aligned}$$

11 $$\begin{aligned}& {}_{0}^{C} D_{t}^{\alpha } \vartheta _{\iota } = \frac{\partial H}{\partial \lambda _{\kappa }},\quad \iota =1,\ldots,6. \end{aligned}$$

Moreover,

12 $$ \lambda _{\iota } ( T_{f} )=0,\quad \iota =1,2,3, \ldots,6. $$

### Remark 1

Under some additional assumptions on the objective functional *J* and the right-hand side of equation (6) must be added, for example, the convexity of *J* and the linearity of *ξ* in $\Psi _{j}$ and $u_{p}$, $u_{ap}$, then the necessary conditions of optimality are also sufficient, for more details, see [27].

### Theorem 4.1

*There exist optimal control variables* $u_{p}^{*}$, $u_{ap}^{*}$*with the corresponding solutions* $S_{p}^{*}$, $E_{p}^{*}$, $I_{p}^{*}$, $A_{p}^{*}$, $R_{p}^{*}$, $M^{*}$*that minimize* $J( u_{p}, u_{ap} )$*over* Ω. *Furthermore*, *there exist adjoint variables* $\lambda _{i}$, $i=1,2,3,\ldots,6$, *satisfy the following*:

(i) *Adjoint equations*:
  13 $$\begin{aligned}& {}_{t}^{C} D_{t_{f}}^{\alpha } \lambda _{1} =\biggl( - \mu _{p}^{\alpha } - \frac{\eta _{p}^{\alpha } I_{p}^{*}}{N_{p}} - \eta _{w}^{\alpha } M \biggr) \lambda _{1} + \biggl( \frac{\eta _{p}^{\alpha } I_{p}^{*}}{N_{p}} + \eta _{w}^{\alpha } M^{*} \biggr) \lambda _{2}, \\& {}_{t}^{C} D_{t_{f}}^{\alpha } \lambda _{2} = - \bigl(\bigl(1 - \theta _{p}^{\alpha } \bigr) w_{p}^{\alpha } + \theta _{p}^{\alpha } \rho _{p}^{\alpha } - \mu _{p}^{\alpha } \bigr) \lambda _{2} + \bigl(\bigl(1 - \theta _{p}^{\alpha } \bigr) w_{p}^{\alpha } \bigr) \lambda _{3} + \theta _{p}^{\alpha } \rho _{p}^{\alpha } \lambda _{4}, \\& {}_{t}^{C} D_{t_{f}}^{\alpha } \lambda _{3} =1 - \frac{\eta _{p}^{\alpha } S_{p}^{*}}{N_{p}} \lambda _{1} + \lambda _{2} \frac{\eta _{p}^{\alpha } S_{p}^{*}}{N_{p}} - \lambda _{3} \bigl( \tau _{p}^{\alpha } + \mu _{p}^{\alpha } + \epsilon _{1} u_{p}^{*} \bigr) + \lambda _{5} \bigl( \tau _{p}^{\alpha } + \epsilon _{1} u_{p}^{*} \bigr) + \lambda _{6} \varrho _{p}^{\alpha }, \\& {}_{t}^{C} D_{t_{f}}^{\alpha } \lambda _{4} =1 - \frac{\eta _{p} \psi ^{\alpha } S_{p}^{*}}{N_{p}} \lambda _{2} - \lambda _{4} \bigl( \tau _{p}^{\alpha } + \mu _{p}^{\alpha } + \epsilon _{2} u_{ap}^{*} \bigr) + \lambda _{5} \bigl( \tau _{ap}^{\alpha } + \epsilon _{2} u_{ap}^{*} \bigr) + \lambda _{6} \varrho _{p}^{\alpha }, \\& {}_{t}^{C} D_{t_{f}}^{\alpha } \lambda _{5} = - \mu _{p}^{\alpha } R^{*} \lambda _{5}, \\& {}_{t}^{C} D_{t_{f}}^{\alpha } \lambda _{6} = - \eta _{w}^{\alpha } S_{p}^{*} \lambda _{1} + \lambda _{2 \eta _{w}^{\alpha } S_{p}^{*}} - \pi ^{\alpha } \lambda _{6}. \end{aligned}$$
(ii) *The transversality conditions*:
  14 $$ \lambda _{\iota } ( T_{f} ) =0,\quad \iota =1,2, \ldots,6. $$
(iii) *Optimality conditions*:
  15 $$\begin{aligned}& H ( S_{p}, E_{p}, I_{p}, A_{p}, R_{p}, M, u_{p}, u_{ap}, \lambda , t ) \\& \quad =\min_{0 \leq u_{p}, u_{ap} \leq 1} H ( S_{p}, E_{p}, I_{p}, A_{p}, R_{p}, M, u_{p}, u_{ap}, \lambda , t ). \end{aligned}$$

*Moreover*,

16 $$\begin{aligned}& u_{p}^{*} =\min \biggl\{ 1,\max \biggl\{ 0, \frac{\epsilon _{1} I_{p}^{*} ( \lambda _{3} - \lambda _{5} )}{B_{1}} \biggr\} \biggr\} , \end{aligned}$$

17 $$\begin{aligned}& u_{ap}^{*} =\min \biggl\{ 1,\max \biggl\{ 0, \frac{\epsilon _{2} A_{p}^{*} ( \lambda _{4} - \lambda _{5} )}{B_{2}} \biggr\} \biggr\} . \end{aligned}$$

### Proof

Equations (13) can be obtained from (9), where

18 $$\begin{aligned} H^{*} =& \lambda _{1}^{C}{}_{a} D_{t}^{\alpha } S_{p}^{*} + \lambda _{2}^{C}{}_{a} D_{t}^{\alpha } E_{p}^{*} + \lambda _{3}^{C}{}_{a} D_{t}^{\alpha } I_{p}^{*} \\ &{}+ \lambda _{4}^{C}{}_{a} D_{t}^{\alpha } A_{p}^{*} + \lambda _{5}^{C}{}_{a} D_{t}^{\alpha } R_{p}^{*} + \lambda _{6}^{C}{}_{a} D_{t}^{\alpha } M^{*} \\ &{}+ I_{p}^{*} ( t ) + A_{p}^{*} ( t ) + \frac{B_{1}}{2} u_{p}^{*} ( t ) + \frac{B_{2}}{2} u_{ap}^{*} ( t ) \end{aligned}$$

is the Hamiltonian. The transversality conditions $\lambda _{\kappa } ( T_{f} )=0$, $\kappa =1,\ldots,6$, hold. Using (15), we can claim Equations (16)–(17).

Now, the state system can be claimed:

19 $$\begin{aligned}& {}_{a}^{C} D_{t}^{\alpha } S_{p}^{*} = \pi _{p}^{\alpha } - \mu _{p}^{\alpha } S_{p}^{*} - \frac{\eta _{p}^{\alpha } ( I_{p}^{*} + \psi ^{\alpha } A_{p}^{*} )}{N_{p}} - \eta _{w}^{\alpha } S_{p}^{*} M^{*}, \\& {}_{a}^{C} D_{t}^{\alpha } E_{p}^{*} = \frac{\eta _{p}^{\alpha } ( I_{p}^{*} + \psi ^{\alpha } A_{p}^{*} )}{N_{p}} + \eta _{w}^{\alpha } S_{p}^{*} M^{*} - \bigl(1 - \theta _{p}^{\alpha } \bigr) w_{p} E_{p}^{*} - \theta _{p}^{\alpha } \rho _{p}^{\alpha } E_{p}^{*}, \\& {}_{a}^{C} D_{t}^{\alpha } I_{p}^{*} =\bigl(1 - \theta _{p}^{\alpha } \bigr) w_{p}^{\alpha } E_{p}^{*} - \bigl( \tau _{p}^{\alpha } + \epsilon _{1} u_{p}^{*} + \mu _{p}^{\alpha } \bigr) I_{p}^{*}, \\& {}_{a}^{C} D_{t}^{\alpha } A_{p}^{*} = \theta _{p}^{\alpha } \rho _{p}^{\alpha } E_{p}^{*} - \bigl( \tau _{ap}^{\alpha } + \epsilon _{2} u_{ap}^{*} + \mu _{p}^{\alpha } \bigr) A_{p}^{*}, \\& {}_{a}^{C} D_{t}^{\alpha } R_{p}^{*} = \tau _{p}^{\alpha } I_{p}^{*} + \epsilon _{1} u_{p}^{*} I_{p}^{*} + \tau _{ap}^{\alpha } A_{p}^{*} + \epsilon _{2} u_{ap}^{*} A_{p}^{*} - \mu _{p}^{\alpha } R_{p}^{*}, \\& {}_{a}^{C} D_{t}^{\alpha } M^{*} = \varrho _{p}^{\alpha } I_{p}^{*} + \varpi _{p}^{\alpha } A_{p}^{*} - \pi ^{\alpha } M^{*}. \end{aligned}$$

 □

## Numerical methods for solving FOCPs

### GL-NWAFDM

In this section, we construct a novel numerical method called GL-NWAFDM as an extension to the method given in [24, 28]. This method can be an explicit method (easy for coding) or an implicit method (more stable and efficient), depending on the weight factor $\Theta \in [0,1]$. To approximate the solutions of system (19) using GL-NWAFDM, we first discretize the Caputo fractional operator (1) with replacing $\triangle ( t )$ by $\varphi ( \triangle t )$, where

$$ \varphi (\triangle t )=\triangle ( t ) +O \bigl(\triangle ( t )^{2} \bigr), \quad 0< \varphi (\triangle t )< 1, \triangle ( t )\longrightarrow 0. $$

Then the discretization for system (19), where $n =0,1,2,3,\ldots, N$, using GL-NWAFDM can be written as follows:

20 $$\begin{aligned}& \begin{aligned} &{S_{p}^{n+1}}^{\ast}-\sum _{i=1}^{n+1} \mu_{i}{S_{p}^{n+1-i}}^{\ast}-q_{n+1}{S_{p}^{0}}^{\ast}\\ &\quad = \Theta \varphi(\triangle t)^{\alpha} \biggl(\pi_{p}^{\alpha} -\mu_{p}^{\alpha}{S^{n+1}_{p}}^{\ast}- \frac{\eta_{p}^{\alpha}({I^{n+1}_{p}}^{\ast}+\psi^{\alpha} {A^{n+1}_{p}}^{\ast})}{N_{p}} -\eta_{w}^{\alpha}{S^{n+1}_{p}}^{\ast}{M^{n+1}}^{\ast}\biggr) \\ &\quad\quad{} +(1-\Theta) \varphi(\triangle t)^{\alpha} \biggl( \pi_{p}^{\alpha} -\mu_{p}^{\alpha}{S^{n}_{p}}^{\ast}- \frac{\eta_{p}^{\alpha}({Ix^{n}_{p}}^{\ast}+\psi^{\alpha} {A^{n}_{p}}^{\ast})}{N_{p}}-\eta_{w}^{\alpha}{S^{n}_{p}}^{\ast}{M^{n}}^{\ast}\biggr), \end{aligned} \\ & \begin{aligned} &{E_{p}^{n+1}}^{\ast}-\sum _{i=1}^{n+1} \mu_{i}{E_{p}^{n+1-i}}^{\ast}-q_{n+1}{E_{p}^{0}}^{\ast}\\ &\quad = \Theta \varphi(\triangle t)^{\alpha} \biggl(\frac{\eta_{p}^{\alpha}({I^{n+1}_{p}}^{\ast}+\psi^{\alpha} {A^{n+1}_{p}}^{\ast})}{N_{p}}+ \eta_{w}^{\alpha}{S^{n+1}_{p}}^{\ast}{M^{n+1}}^{\ast}\\ &\quad\quad{} -\bigl(1-\theta_{p}^{\alpha}\bigr)w_{p}^{\alpha}{E^{n+1}_{p}}^{\ast}- \theta_{p}^{\alpha}\rho_{p}^{\alpha}{E^{n+1}_{p}}^{\ast}\biggr) \\ &\quad\quad{} +(1-\Theta)\varphi(\triangle t)^{\alpha} \biggl(\frac{\eta_{p}^{\alpha}({I^{n}_{p}}^{\ast}+\psi^{\alpha} {A^{n}_{p}}^{\ast})}{N_{p}}+ \eta_{w}^{\alpha}{S^{n}_{p}}^{\ast}{M^{n}}^{\ast}-(1-\theta_{p})w_{p}^{\alpha}{E^{n}_{p}}^{\ast}- \theta_{p}^{\alpha}\rho_{p}^{\alpha}{E^{n}_{p}}^{\ast}\biggr), \end{aligned} \\ & \begin{aligned} &{I_{p}^{n+1}}^{\ast}-\sum _{i=1}^{n+1}\mu_{i}{I_{p}^{n+1-i}}^{\ast}-q_{n+1}{I_{p}^{0}}^{\ast}\\ &\quad = \Theta \varphi(\triangle t)^{\alpha} \bigl(\bigl(1-\theta_{p}^{\alpha} \bigr)w_{p}^{\alpha}{E^{n+1}_{p}}^{\ast}- \bigl(\tau_{p}^{\alpha}+\epsilon_{1}{u^{n+1}_{p}}^{\ast}+ \mu_{p}^{\alpha}\bigr) {I^{n+1}_{p}}^{\ast}\bigr) \\ &\quad\quad{} +(1-\Theta)\varphi(\triangle t)^{\alpha}\bigl(\bigl(1- \theta_{p}^{\alpha}\bigr)w_{p}^{\alpha}{E^{n}_{p}}^{\ast}- \bigl(\tau_{p}^{\alpha}+\epsilon_{1}{u^{n}_{p}}^{\ast}+ \mu_{p}^{\alpha}\bigr) {I^{n}_{p}}^{\ast}\bigr), \end{aligned} \\ & \begin{aligned} & {A_{p}^{n+1}}^{\ast}-\sum _{i=1}^{n+1} \mu_{i}{A_{p}^{n+1-i}}^{\ast}-q_{n+1}{A_{p}^{0}}^{\ast}\\ &\quad = \Theta\varphi (\triangle t)^{\alpha} \biggl(\frac{\eta_{p}^{\alpha}({I^{n+1}_{p}}^{\ast}+\psi^{\alpha} {A^{n+1}_{p}}^{\ast})}{N_{p}}+ \eta_{w}^{\alpha}{S^{n+1}_{p}}^{\ast}{M^{n+1}}^{\ast}\\ &\quad\quad{} -\bigl(1-\theta_{p}^{\alpha}\bigr)w_{p}^{\alpha}{E^{n+1}_{p}}^{\ast}- \theta_{p}^{\alpha}\rho_{p}^{\alpha}{E^{n+1}_{p}}^{\ast}\biggr) \\ &\quad\quad{} +(1-\Theta)\varphi (\triangle t)^{\alpha} \biggl( \frac{\eta_{p}^{\alpha}({I^{n}_{p}}^{\ast}+\psi^{\alpha} {A^{n}_{p}}^{\ast})}{N_{p}}+\eta_{w}{S^{n}_{p}}^{\ast}{M^{n}}^{\ast}-(1-\theta_{p})w_{p}^{\alpha}{E^{n}_{p}}^{\ast}- \theta_{p}\rho_{p}^{\alpha}{E^{n}_{p}}^{\ast}\biggr), \end{aligned} \\ & {R_{p}^{n+1}}^{\ast}-\sum _{i=1}^{n+1} \mu_{i}{R_{p}^{n+1-i}}^{\ast}-q_{n+1}{R_{p}^{0}}^{\ast} \\ & \quad = \Theta \varphi(\triangle t)^{\alpha} \bigl(\tau_{p}^{\alpha}{I^{n+1}_{p}}^{\ast}+ \epsilon_{1}{u^{n+1}_{p}}^{\ast}{I^{n+1}_{p}}^{\ast}+\tau_{ap}^{\alpha}{A^{n+1}_{p}}^{\ast}+\epsilon_{2}{u^{n+1}_{ap}}^{\ast}{A^{n+1}_{p}}^{\ast}-\mu_{p}^{\alpha}{R^{n+1}_{p}}^{\ast}\bigr) \\ & \quad\quad{} +(1-\Theta) \varphi(\triangle t)^{\alpha} \bigl(\tau_{p}^{\alpha}{I^{n}_{p}}^{\ast}+ \epsilon_{1}{u^{n}_{p}}^{\ast}{I^{n}_{p}}^{\ast}+\tau_{ap}^{\alpha}{A^{n}_{p}}^{\ast}+\epsilon_{2}{u^{n}_{ap}}^{\ast}{A^{n}_{p}}^{\ast}-\mu_{p}^{\alpha}{R^{n}_{p}}^{\ast}\bigr), \\ & \begin{aligned}[b] {M^{n+1}}^{\ast}-\sum_{i=1}^{n+1} \mu_{i}{M^{ n+1-i}}^{\ast}-q_{n+1}{M^{ 0}}^{\ast}&= \Theta \varphi(\triangle t)^{\alpha} \bigl(\varrho_{p}^{\alpha}{I^{n+1 }_{p}}^{\ast}+ \varpi_{p}^{\alpha}{A_{p}^{n+1}}^{\ast}- \pi^{\alpha} {M^{n+ }}^{\ast}\bigr) \\ &\quad{} +(1-\Theta) \varphi(\triangle t)^{\alpha} \bigl(\varrho_{p}^{\alpha}{I^{n+1}_{p}}^{\ast}+ \varpi_{p}^{\alpha}{A^{n}_{p}}^{\ast}- \pi^{\alpha} \bigr). \end{aligned} \end{aligned}$$

This system is a nonlinear algebraic system of $(6 N+ 6)$ equation of $(6 N+ 6)$ unknown $( S_{p}^{*}, E_{p}^{*}, I_{p}^{*}, A_{p}^{*}, R_{p}^{*}, M^{*} )$, that can be solved using an appropriate iterative method depending on the supposed initial conditions. We notice that this scheme is explicit for $\Theta =0$, partially implicit for $0<\Theta <1$, and fully implicit when $\Theta =1$.

### Stability of GL-NWAFDM

In the following we show that the GL-NWAFDM in case $0<\Theta \leq 1$ (implicit case) is unconditionally stable. In order to investigate the stability of the proposed method when $(\Theta \neq 0)$, consider the model test problem of linear fractional differential equation

21 $$ \bigl({}_{a}^{c} D_{t}^{\alpha } \bigr) f ( t )= \text{Af} ( t ),\quad t >0, 0< \alpha \leq 1, A < 0. $$

Let $f ( t_{n} )= f_{n} = \zeta _{n}$ be the approximate solution of this equation, then using GL-NWAFDM with (1) we rewrite equation (21) in the following form:

$$ \zeta ^{n+ 1} - \sum_{i =1}^{n+ 1} \mu _{i} \zeta ^{n+ 1 -i} - q_{n+ 1} \zeta ^{0} = \varphi (\triangle t )^{\alpha } \bigl(\Theta A \zeta ^{n+ 1} + (1 - \Theta ) A \zeta ^{n} \bigr). $$

Then we have

$$ \zeta ^{n+ 1} = \frac{1}{(1 -\varphi (\triangle t )^{\alpha } \Theta A )} \Biggl( \sum _{i =1}^{n+ 1} \mu _{i} \zeta ^{n+ 1 -i} + q_{n+ 1} \zeta ^{0} + (1 - \Theta ) \varphi (\triangle t )^{\alpha } A \zeta ^{n} \Biggr),\quad n \geq 1, $$

we have $\frac{1}{(1 -\varphi (\triangle t )^{\alpha } \Theta A )} <1$, hence

$$\begin{aligned}& \zeta ^{1} \leq \zeta ^{0}, \\& \zeta ^{n+ 1} \leq \zeta ^{n} \leq \zeta ^{n- 1} \cdots \zeta ^{1} \leq \zeta ^{0}. \end{aligned}$$

So the proposed implicit scheme is stable.

## Numerical simulations

**Figure 1:**
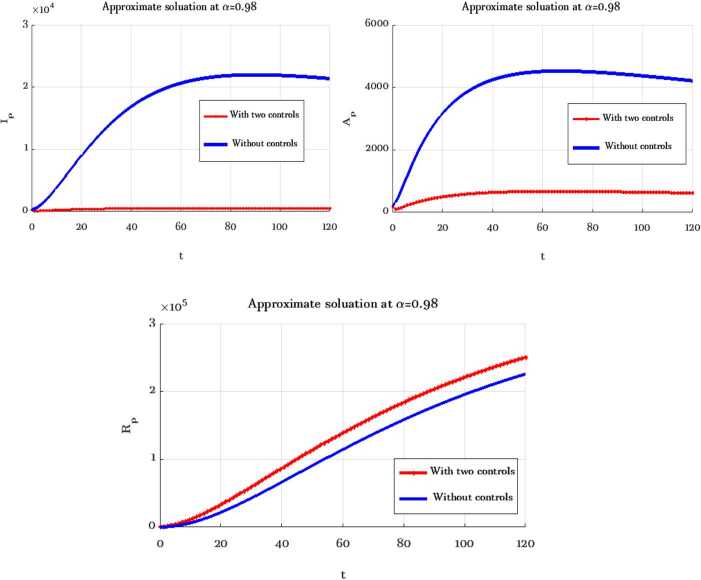
Numerical simulations of $I_{p}$, $A_{p}$, and $R_{p}$ at $\alpha =0.98$, $\Theta =1$ with and without controls

**Figure 2:**
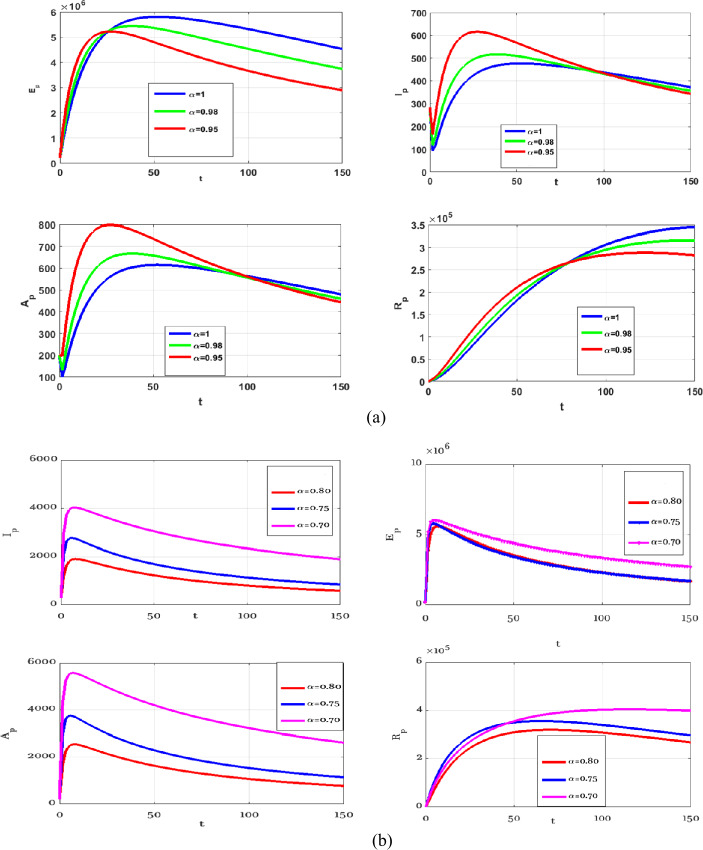
Numerical simulations at different *α*, $\Theta =1$, $B_{1} = B_{2} =500$, and $\epsilon _{1} = \epsilon _{2} =10$ with two controls

**Figure 3:**
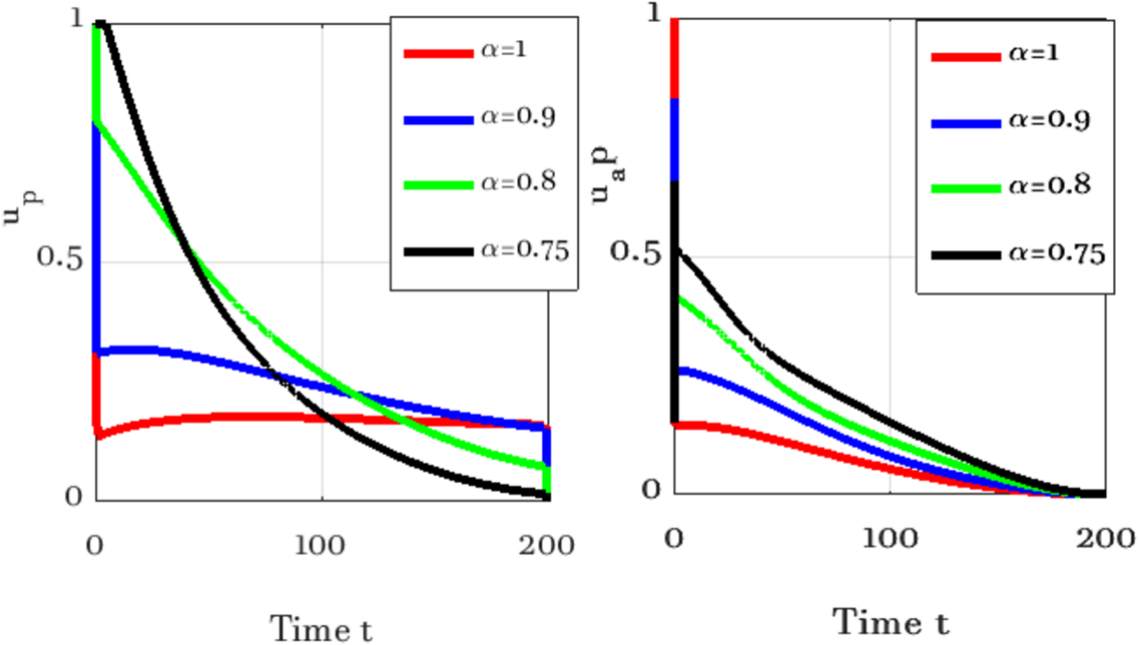
Numerical simulations of $u_{p}$, $u_{ap}$ at different *α*, $\Theta =1$

**Figure 4:**
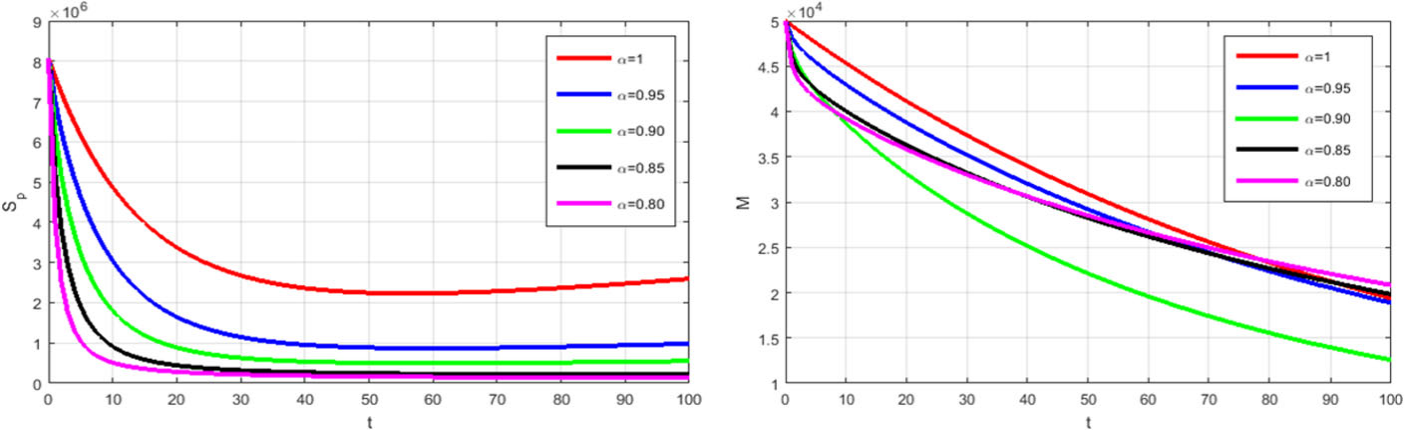
Numerical simulations of $S_{p}$, *M* at different *α*, $\Theta =1$

**Figure 5:**
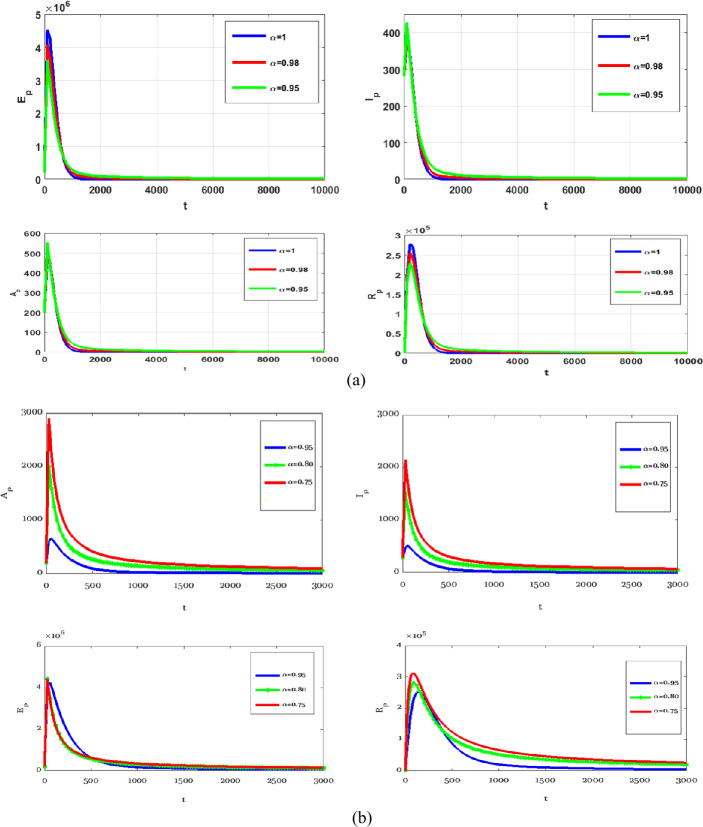
Numerical simulations of $E_{p}$, $I_{p}$, $A_{p}$, and $R_{p}$ at different *α*, $\Theta =1$, $T_{f} =3000, 10{,}000$ with controls

In this section, numerical simulations of the proposed model (19) and (13) with and without optimal control are presented. Gl-NWAFDM is given to obtain the numerical results of the state equations (19) with the following initial conditions [4]: $S_{p} (0)=8{,}065{,}518$, $E_{p} (0)=200{,}000$, $I_{p} (0)=282$, $A_{p} (0)=200$, $R_{p} (0)=0$, $M (0)=50{,}000$, and we consider $N_{p} =8{,}266{,}000$. Then, by using the implicit finite difference method [21], we solve (13) with (14) by using different values of $0< \alpha \leq 1$ with $B_{1} = B_{2} =500$, $\epsilon _{1} = \epsilon _{2} =10$, and $0<\Theta \leq 1$. In the numerical simulations the time level is chosen in days, it is up to $10{,}000$. The graphical results obtained through these figures demonstrate that in the case without control, the number of the infected and the asymptotically infected population is increasing, while the number of the population is decreasing in the controlled case as we can see in Fig. 1. This figure demonstrates the effectiveness of two control cases for the proposed model. Moreover, Table 3 reports the values of the objective functional obtained by the proposed method with and without controls and $\Theta =1,0.5$. We note that the results obtained in the case of fully implicit at ($\Theta =1$) are better than the results in the case $\Theta =0.5$. Also, the best result of the control case is given at $\alpha =0.8$. Figure 2 shows how the behavior of the solutions in the control case is changing by using different values of *α* and $T_{f} =150$. Figure 3 shows the evolution of the approximate solutions for the control variables using different *α*. It is noted that the range of the solution remains between zero and one. The approximate solutions for $S_{p}$ and *M* with control case and different values of *α* are given in Fig. 4. Figure 5 illustrates the behavior of the approximate solutions of $E_{p}$, $I_{p}$, $A_{p}$, $R_{p}$ at different values of *α* at big time, and it is demonstrated that the proposed method is unconditionally stable at $\Theta =1$. The results are given by using MATLAB (R2015a).

**Table 3:** GL-NWAFDM results of cost functional without and with controls, $T_{f} =120$ and $\phi (\triangle t )=(1 - e^{- \triangle t} )$ and different Θ, *α*

*α*	$J ( u_{p}^{*}, u_{ap}^{*} )$ without control	*J* with 2 controls	*J* with 2 controls
		Θ = 1	Θ = 0.5
1	2.3111 × 10^6^	3.3868 × 10^3^	3.6001 × 10^3^
0.97	2.5948 × 10^6^	3.2618 × 10^3^	3.6290 × 10^3^
0.8	5.4554 × 10^6^	2.9671 × 10^3^	3.5030 × 10^3^
0.7	8.9249 × 10^6^	3.3427 × 10^3^	5.9119 × 10^3^
0.6	2.5717 × 10^7^	3.6939 × 10^3^	5.0904 × 10^3^
0.5	2.1879 × 10^7^	3.2873 × 10^3^	4.7705 × 10^3^

## Conclusions

In the present work, the optimal control of coronavirus model with fractional operator is presented. Also, the combination of fractional order derivative and optimal control in the model improves the dynamics and increases the complexity of the model. Two control variables, $u_{p} ( t )$ and $u_{ap} ( t )$, are added to health care such as isolating patients in private health rooms and providing respirators and giving them treatments soothing regularly. These have been implemented to minimize the number of the infected and the asymptotically infected as we can see in Fig. 1. Necessary optimality conditions are derived. GL-NWAFDM is constructed to study the behavior of the proposed problem. This method depends on the values of the factor Θ. It can be explicit or implicit with large stability regions as we can see in our results. Moreover, the stability analysis of the proposed method is studied. It was shown that this method has good stability properties in the implicit case. Some simulations are presented to support our theoretical findings. It is concluded that GL-NWAFDM can be applied to solve such fractional optimality systems simply and effectively.
